# Suspected NIUAA after indobufen in a patient with ibuprofen allergy: a case report suggesting possible cross-reactivity

**DOI:** 10.3389/fphar.2026.1932735

**Published:** 2026-09-03

**Authors:** Long Luo, Jun Li, Ying Yuan, Ling Zhu

**Affiliations:** Department of Neurology, The Central Hospital of Xiangtan (The Affiliated Hospital of Hunan University), Xiangtan, Hunan, China

**Keywords:** case report, cross-reactivity, ibuprofen, indobufen, non-steroidal anti-inflammatory drugs

## Abstract

Non-steroidal anti-inflammatory drugs (NSAIDs) are commonly used in clinical practice, and hypersensitivity reactions to them are not uncommon. Although ibuprofen and indobufen have different clinical indications, both exert their effects through cyclooxygenase inhibition, suggesting a potential risk of cross-reactivity. This report describes a 52-year-old female patient who experienced pruritus, facial and eyelid edema, tearing, and ocular itching approximately half an hour after two separate exposures to ibuprofen in the past. During the current hospitalization for cerebral infarction, she received indobufen for antiplatelet therapy and developed similar symptoms—eyelid edema, tearing, and pruritus of the head and face—within approximately 10 min. The suspected drug was discontinued and antihistamine therapy was administered, following which the symptoms resolved. Subsequent continued use of other concomitant medications, along with antiplatelet therapy with clopidogrel and cilostazol, did not elicit any allergic reactions; this case suggests indobufen as a possible culprit allergen, and its shared COX-1 inhibitory mechanism with ibuprofen—despite their different chemical groups—raises the possibility of cross-reactive hypersensitivity between the two agents, although this inference remains to be confirmed. Although allergic reactions to indobufen are rare in clinical practice, clinicians should remain vigilant about the potential risk of cross-reactivity when prescribing indobufen to patients with a history of NSAID hypersensitivity.

## 1 Introduction

Indobufen is an isoindolinyl phenylbutyric acid derivative that has gained increasing use in the prevention and treatment of cardiovascular and cerebrovascular diseases in recent years. As a commonly used antiplatelet agent, indobufen exerts its antiplatelet aggregation effect through reversible inhibition of platelet cyclooxygenase, thereby reducing the production of thromboxane B2 (Bhana and McClellan, 2001). This mechanism is shared with non-steroidal anti-inflammatory drugs (NSAIDs).

NSAIDs are a common cause of drug hypersensitivity reactions. Epidemiological studies have shown that the prevalence of self-reported NSAID hypersensitivity in the general adult population ranges from 1.9% to 3.5% (Gomes et al., 2004; Zhou et al., 2016). Ibuprofen, a propionic acid derivative, is a frequently implicated NSAID, with an incidence second only to aspirin (Nissen et al., 2015). Approximately 76% of NSAID hypersensitivity reactions are cross-reactive, meaning that patients react to multiple NSAIDs with different chemical groups (Doña et al., 2011; Miniello et al., 2024; Romano et al., 2025). The mechanism primarily involves COX-1 inhibition (Doña et al., 2020).

This case report presents a patient with a history of ibuprofen allergy and a suspected allergic reaction to indobufen, aiming to draw clinicians’ attention to the potential for cross-reactivity to NSAIDs, particularly the possible risk associated with indobufen when used as an antiplatelet agent.

## 2 Case report

A 52-year-old female patient presented with a 4-day history of involuntary choreiform movements of the right upper and lower limbs, which occurred during wakefulness and disappeared during sleep, accompanied by gait disturbance. Her mental status remained clear throughout, with no limb numbness, weakness, headache, vomiting, blurred vision, diplopia, vertigo, or incontinence. There was no recent history of polydipsia, polyuria, weight loss, fever, pharyngalgia, diarrhea, or emotional disturbance. Her past medical history included hypertension (managed with levamlodipine 2.5 mg once daily), appendectomy, and dental root canal treatment. She had no history of exposure to toxic substances and no family history of genetic disorders. Notably, she reported a history of ibuprofen allergy: on two separate occasions, approximately half an hour after taking ibuprofen, she developed bilateral eyelid swelling, tearing, and skin pruritus, which resolved spontaneously after drug discontinuation. She denied any other drug or food allergies.

On admission, her blood pressure was 155/96 mmHg. Neurological examination revealed clear consciousness and fluent speech, no cranial nerve abnormalities, normal muscle strength (grade V) and symmetric muscle tone in all four limbs, choreiform involuntary movements of the right limbs, bilateral negative Babinski signs, normal sensory function, and intact coordination. Gait was mildly unsteady. Laboratory investigations showed elevated triglycerides (12.36 mmol/L), while complete blood count, urinalysis, stool routine, liver and kidney function, cardiac enzymes, electrolytes, homocysteine, thyroid function tests, ceruloplasmin, and glycated hemoglobin were all essentially within normal ranges (see Supplementary Table S1 for details). Non-contrast head CT showed no hemorrhage or space-occupying lesions, and electrocardiography was normal.

A diagnosis of acute ischemic stroke was suspected. On the day of admission, she was started on indobufen (100 mg twice daily), atorvastatin calcium (20 mg once nightly), fenofibrate (200 mg once daily), levamlodipine (2.5 mg once daily), and ginkgo biloba extract. Approximately 10 min after the first dose of indobufen, she developed bilateral ocular fullness, foreign body sensation (predominantly in the left eye), tearing, and pruritus of the head and face. Physical examination revealed pronounced edematous maculopapular rash on the face and bilateral eyelids. Ophthalmology consultation suggested allergic eyelid edema and allergic conjunctivitis, and dermatology consultation diagnosed allergic dermatitis. Indobufen was immediately discontinued as the suspected culprit drug, while other medications were continued. Antihistamine therapy was initiated with oral ebastine (10 mg once daily), intravenous calcium gluconate (1 g), and intravenous dexamethasone (5 mg). Her ocular and cutaneous symptoms gradually resolved within 24 h. Subsequent continued use of the other medications without recurrence of allergy supported indobufen as the most likely causative agent.

During hospitalization, brain MRI revealed an acute lacunar infarct in the left corona radiata and parietal lobe, with severe stenosis/near-occlusion of the M1 segment of the left middle cerebral artery. The etiology of the stroke was considered to be large artery atherosclerosis. Laboratory findings did not support thyrotoxicosis, Wilson’s disease, or diabetic non-ketotic hemichorea, and there was no history of infection, toxic exposure, or hereditary disease. The involuntary movements were attributed to the new-onset left parietal infarct, which has been reported to cause contralateral involuntary limb movements (Rossetti et al., 2003; Patel et al., 2019). Given the patient’s history of ibuprofen allergy and the suspected cross-reactive reaction to indobufen, aspirin was not attempted. The antiplatelet regimen was switched to clopidogrel (75 mg once daily) combined with cilostazol (100 mg twice daily). No further allergic reactions occurred during the remainder of her hospitalization, and she was discharged in stable condition.

According to the Naranjo Adverse Drug Reaction Probability Scale, the score for indobufen was 8, indicating a probable causal relationship (Doña et al., 2020) (see Supplementary Table S2 for details). According to the modified Hartwig and Siegel Severity Assessment Scale, the severity was classified as level 3 (required discontinuation of the suspected drug and symptomatic treatment, without prolongation of hospitalization) (Hartwig et al., 1992) (see Supplementary Table S3 for details).

## 3 Discussion

In the present case, there was a clear temporal association between indobufen and the hypersensitivity reaction: symptoms appeared approximately 10 min after the first dose and resolved rapidly upon drug discontinuation. Notably, among the concomitant medications, levamlodipine had been previously well tolerated by the patient; although atorvastatin, fenofibrate, and Ginkgo biloba extract had not been previously taken by the patient, they were continued after discontinuation of indobufen and did not provoke any allergic reactions, strongly suggesting indobufen as the most likely culprit drug. The Naranjo Adverse Drug Reaction Probability Scale score was +8, indicating a “probable” causal relationship. This score was based on the following factors: previous literature reports of indobufen-induced cutaneous allergic reactions (+1) (Li et al., 2025); the adverse event occurred within minutes after drug administration (+2); symptoms improved rapidly after drug discontinuation and antihistamine therapy (+1); no alternative causes were identified (+2); the patient had a prior history of similar reactions to ibuprofen (+1); and the reaction was confirmed by ophthalmology and dermatology consultations (+1). Nevertheless, it should be acknowledged that the score has certain limitations—the improvement of symptoms was potentially confounded by the concurrent use of corticosteroids and antihistamines, concomitant medications also represent a potential confounding factor (although no abnormalities were observed with continued use after withdrawal), and rechallenge with indobufen was not performed. Therefore, this cross-reactivity conclusion should be regarded as a reasonable inference based on the available clinical evidence, rather than definitive proof.

The potential for cross-reactivity between ibuprofen and indobufen has not been previously described. Ibuprofen is a common cause of NSAID hypersensitivity (Podlecka et al., 2023), typically presenting with rapid onset (median latency 30 min) and angioedema as the predominant manifestation (90%). Age has been identified as a significant risk factor (adjusted odds ratio 1.46 per year of age) (Mori et al., 2020). In contrast, indobufen is a commonly used antiplatelet agent, and allergic reactions to it are rarely reported. Li et al., 2025 first reported a case of cross-reactivity between aspirin and indobufen presenting as drug-induced hypersensitivity syndrome (DIHS) (Li et al., 2025).

NSAID hypersensitivity reactions are classified as either cross-reactive, mediated by cyclooxygenase inhibition, or selective, mediated by specific IgE antibodies or T cells. Ibuprofen (a propionic acid derivative) and indobufen (an isoindolinyl phenylbutyric acid derivative) belong to different chemical groups, and the reaction was presumed to be a cross-reactive (mediated by cyclooxygenase inhibition) NSAID hypersensitivity, which manifests as three main clinical phenotypes: N-ERD in patients with underlying chronic respiratory disease, NECD in those with a history of chronic urticaria, and NIUAA in otherwise healthy individuals (Romano et al., 2025). According to this updated classification, the clinical presentation of this patient fits best with the diagnostic framework of NIUAA: an immediate reaction (≤6 h) to two NSAIDs of different chemical groups (ibuprofen and indobufen) (Kowalski et al., 2013), manifesting as eyelid edema, tearing, and skin pruritus involving both the skin and mucous membranes—a typical “blended” reaction—without underlying chronic spontaneous urticaria, asthma, or sinus disease. However, without confirmatory testing, a definitive classification cannot be made.

The pathophysiological mechanism underlying cross-reactive NSAID hypersensitivity centers on the reduction of prostaglandin E2 (PGE2) production following COX-1 inhibition. Under normal physiological conditions, PGE2 exerts protective effects by inhibiting eosinophil migration and mast cell degranulation via the EP2 receptor, thereby reducing circulating levels of histamine and tryptase, as well as by suppressing 5-lipoxygenase translocation to the nuclear membrane, thus decreasing leukotriene production (Miniello et al., 2024). When COX-1 is inhibited, PGE2 production is significantly reduced, removing these protective mechanisms. In susceptible individuals, PGE2 depletion leads to overactivation of the 5-lipoxygenase pathway, excessive leukotriene generation, and enhanced mast cell activation with release of histamine and other pro-inflammatory mediators (Miniello et al., 2024; Singh et al., 2013). Because the mechanism of this hypersensitivity is COX-1 inhibition rather than an immune response specific to a particular drug, affected patients typically exhibit cross-reactivity to multiple NSAIDs with COX-1 inhibitory activity (Miniello et al., 2024).

One of the core mechanisms of indobufen is reversible inhibition of COX-1, which reduces thromboxane A2 (TXA2) production and exerts antiplatelet aggregation effects. Therefore, it theoretically carries a potential risk of cross-reactivity with other non-selective NSAIDs. However, clinicians are often insufficiently aware of this potential, and the risk of allergic reactions to indobufen is frequently overlooked. For patients with a clear history of ibuprofen or other NSAID hypersensitivity, or those with asthma or chronic urticaria (in whom the incidence of NSAID hypersensitivity is approximately 30%) (Pham et al., 2016), the first dose of COX-1 inhibitor antiplatelet agents (e.g., indobufen or aspirin) is recommended to be administered in a healthcare setting to mitigate the risk of cross-reactivity. If an allergic reaction occurs, the drug should be discontinued immediately, and antihistamines, corticosteroids, or epinephrine should be administered according to the severity of the reaction. For such patients, antiplatelet agents that do not depend on the COX-1 pathway—such as P2Y12 receptor inhibitors (clopidogrel, ticagrelor) or the selective phosphodiesterase III inhibitor cilostazol—may be preferred as alternatives to avoid the risk of cross-reactivity. In cases where no alternative to a COX-1 inhibitor is available and its use is deemed essential, individualized tolerance assessment—including drug provocation testing, desensitization, or *in vitro* testing—may be considered under the guidance of a specialist physician. Another issue worth attention is the tolerability of selective COX-2 inhibitors when these patients require anti-inflammatory or analgesic therapy for other reasons (e.g., arthritis or postoperative pain). Data indicate that the reaction rate to parenteral parecoxib in provocation tests among cross-reactive patients is extremely low, with some studies reporting 0% (Colanardi et al., 2008). Celecoxib is generally well tolerated, with a reported reaction rate of approximately 3.5% in a literature review (Li et al., 2019), while the rates for etoricoxib, meloxicam, nimesulide, rofecoxib, and valdecoxib are somewhat higher (Li and Laidlaw, 2019; Çelik et al., 2013; Terzioğlu et al., 2020; Sánchez-Borges et al., 2005). Therefore, the risk of hypersensitivity should be carefully assessed before clinical use, and drug provocation testing under close monitoring should be considered when necessary to confirm individual tolerance.

## 4 Limitations

This case has several limitations. First, drug provocation testing was not performed to definitively confirm the causal relationship between indobufen and the hypersensitivity reaction, due to ethical and safety concerns. Second, other confirmatory tests that could directly identify the offending agent (such as basophil activation test, skin testing, and graded drug provocation testing), as well as surrogate biomarkers that could indirectly reflect the mechanisms of hypersensitivity (such as serum tryptase and urinary leukotriene E4), are of great value in identifying the culprit allergen. These investigations were not performed in the present case, partly because several of these tests are not available at our institution, and partly due to safety concerns. Third, the conclusions drawn from a single case report require further validation through additional studies.

## 5 Conclusion

This case suggests indobufen as a possible culprit allergen in this patient. Based on its shared COX-1 inhibitory mechanism with ibuprofen and their distinct chemical groups, it further suggests a possible cross-reactive hypersensitivity reaction between the two agents. The NIUAA diagnostic framework could be applied to explain the patient’s clinical presentation; however, the above inference remains to be confirmed by further validation. Although allergic reactions to indobufen are rarely reported, clinicians should remain vigilant in patients with a history of NSAID hypersensitivity, as well as those with comorbid asthma or chronic urticaria, and should carefully elicit drug allergy history and strengthen medication monitoring when prescribing indobufen or aspirin. For such patients, antiplatelet agents that do not depend on the COX-1 pathway—such as clopidogrel, ticagrelor, or cilostazol—may be preferred as alternative choices.

## Data Availability

The original contributions presented in the study are included in the article/Supplementary Material, further inquiries can be directed to the corresponding author.

## Supplementary material

Supplementary Table S1 Laboratory findings.

Supplementary Table S2 Naranjo adverse drug reaction probability scale.

Supplementary Table S3 Hartwig and Siegel’s Severity Assessment Scale.
