# Supplementary material for: Suspected NIUAA after indobufen in a patient with ibuprofen allergy: a case report suggesting possible cross-reactivity
Source: Front Pharmacol. 2026 Sep 3;17:1932735. doi: 10.3389/fphar.2026.1932735 (PMC13581743; doi:10.3389/fphar.2026.1932735)
Supplement: Supplementary file 1 [file Table1.docx]

Supplementary Table 1 laboratory findings

| **Test** | **Result** | **Reference Range** |
| --- | --- | --- |
| **Routine blood tests** |  |  |
| White blood cell count (WBC) | 7.89 × 10⁹/L | 3.69–9.16 × 10⁹/L |
| Neutrophil count (NEU) | 4.74 × 10⁹/L | 2.0–7.0 × 10⁹/L |
| Neutrophil percentage (NEU%) | 60.0% | 50.0–70.0% |
| Lymphocyte count (LYM) | 2.5 × 10⁹/L | 0.8–4.0 × 10⁹/L |
| Lymphocyte percentage (LYM%) | 31.6% | 20.0–40.0% |
| Eosinophil count (EOS) | 0.14 × 10⁹/L | 0.02–0.50 × 10⁹/L |
| Eosinophil percentage (EOS%) | 1.8% | 0.5–5.0% |
| Hemoglobin (HGB) | 132.0 g/L | 110–150 g/L |
| Platelet count (PLT) | 237.0 × 10⁹/L | 101.0–320.0 × 10⁹/L |
| Mean platelet volume (MPV) | 11.8 fL ↑ | 7.2–11.1 fL |
| **Biochemical tests** |  |  |
| Alanine aminotransferase (ALT) | 15.6 IU/L | 0.0–40.0 IU/L |
| Aspartate aminotransferase (AST) | 17.5 IU/L | 0.0–45.0 IU/L |
| Creatinine (CREA) | 47.0 μmol/L | 53.0–115.0 μmol/L |
| Urea | 5.40 mmol/L | 2.0–8.0 mmol/L |
| Uric acid (UA) | 283.0 μmol/L | 89.0–430.0 μmol/L |
| Glucose (GLU) | 6.05 mmol/L | 3.90–6.10 mmol/L |
| Triglycerides (TG) | 12.36 mmol/L ↑ | < 1.70 mmol/L |
| Total cholesterol (CHOL) | 5.13 mmol/L | 2.90–5.20 mmol/L |
| High-density lipoprotein (HDL) | 2.08 mmol/L | > 1.04 mmol/L |
| Low-density lipoprotein (LDL) | 2.74 mmol/L↑ | < 1.4 mmol/L (for stroke patients) |
| Homocysteine (HCY) | 5.70 μmol/L | 4.0–15.4 μmol/L |
| Lactate dehydrogenase (LDH) | 330.0 IU/L ↑ | 114.0–240.0 IU/L |
| Creatine kinase (CK) | 166.0 IU/L | 25.0–195.0 IU/L |
| Creatine kinase-MB (CK-MB) | 24 IU/L | 2.0–25.0 IU/L |
| C-reactive protein (CRP) | Negative | Negative |
| **Coagulation function** |  |  |
| Prothrombin time (PT) | 11.9 s | 11.0–14.5 s |
| International normalized ratio (INR) | 0.88 | 0.8–1.25 |
| Activated partial thromboplastin time (APTT) | 33.8 s | 28.0–43.0 s |
| Fibrinogen (FIB) | 3.41 g/L | 2.00–4.00 g/L |
| D-dimer | 0.25 μg/mL | 0.00–0.70 μg/mL |
| **Thyroid function tests** |  |  |
| Free triiodothyronine (FT3) | 4.05 pmol/L | 3.10–6.80 pmol/L |
| Free thyroxine (FT4) | 12.80 pmol/L | 12.00–22.00 pmol/L |
| Thyroid-stimulating hormone (TSH) | 1.98 μIU/mL | 0.27–4.20 μIU/mL |
| **Other tests** |  |  |
| Glycated hemoglobin (HbA1c) | 5.7% | 4.2–5.9% |
| Ceruloplasmin (CER) | 0.333 g/L | 0.200–0.600 g/L |
| **Urinalysis** |  |  |
| Occult blood | (+) | Negative |
| Protein | Negative | Negative |
| Red blood cells (microscopy) | 0 /HP | 0–3 /HP |
| White blood cells (microscopy) | 0 /HP | 0–8 /HP |
| **Stool routine** |  |  |
| Occult blood | Negative | Negative |
| White blood cells (microscopy) | 0–1 /HP | 0–1 /HP |
