# Supplementary material for: Suspected NIUAA after indobufen in a patient with ibuprofen allergy: a case report suggesting possible cross-reactivity
Source: Front Pharmacol. 2026 Sep 3;17:1932735. doi: 10.3389/fphar.2026.1932735 (PMC13581743; doi:10.3389/fphar.2026.1932735)
Supplement: Supplementary file 2 [file Table2.docx]

| Naranjo adverse drug reaction probability scale | | | | |
| --- | --- | --- | --- | --- |
| Question | Yes | No | Do not know | Score |
| 1.Are there previous conclusive reports on this reaction? | +1 | 0 | 0 | +1 |
| 2.Did the adverse event appear after the suspected drug was administered? | +2 | -1 | 0 | +2 |
| 3. Did the adverse reaction improve when the drug was discontinued or a specific antagonist was administered? | +1 | 0 | 0 | +1 |
| 4.Did the adverse reaction reappear when the drug was readministered? | +2 | -1 | 0 | 0 |
| 5.Are there alternative causes (other than the drug) that could on their own have caused the reaction? | -1 | +2 | 0 | +2 |
| 6.Did the reaction reappear when a placebo was given? | -1 | +1 | 0 | 0 |
| 7.Was the drug detected in the blood (or other fluids) in concentrations known to be toxic? | +1 | 0 | 0 | 0 |
| 8. Was the reaction more severe when the dose was increased or less severe when the dose was decreased? | +1 | 0 | 0 | 0 |
| 9. Did the patient have a similar reaction to the same or similar drugs in any previous exposure? | +1 | 0 | 0 | +1 |
| 10. Was the adverse event confirmed by any objective evidence? | +1 | 0 | 0 | +1 |
| TOTAL SCORE: |  |  |  | +8 |

The Naranjo adverse drug reaction probability scale score was calculated as **+8**, indicating a **probable** association between the adverse reaction and indobufen. This score was based on the following factors: previous literature reports of indobufen-induced cutaneous allergic reactions (+1); the adverse event occurred within minutes after drug administration (+2); symptoms improved rapidly after drug discontinuation and antihistamine therapy (+1); no alternative causes were identified (+2); the patient had a prior history of similar reactions to ibuprofen (+1); and the reaction was confirmed by ophthalmology and dermatology consultations (+1).
