# Supplementary material for: Suspected NIUAA after indobufen in a patient with ibuprofen allergy: a case report suggesting possible cross-reactivity
Source: Front Pharmacol. 2026 Sep 3;17:1932735. doi: 10.3389/fphar.2026.1932735 (PMC13581743; doi:10.3389/fphar.2026.1932735)
Supplement: Supplementary file 3 [file Table3.docx]

**Hartwig and Siegel’s severity assessment scale.**

Mild: Level 1 or 2, Moderate: Level 3 or 4, Severe: Level 5, 6 or 7.

| Hartwig and Siegel’s Severity Assessment Scale | |
| --- | --- |
| Level 1 | An ADR occurred but required no change in treatment with the suspected drug |
| Level 2 | The ADR required that treatment with the suspected drug be held, discontinued, or otherwise changed. No antidote or other treatment requirement was required. No increase in length of stay (LOS) |
| Level 3 | The ADR required that treatment with the suspected drug be held, discontinued, or otherwise changed. AND/OR An Antidote or other treatment was required. No increase in length of stay (LOS) |
| Level 4 | Any level 3 ADR which increases the length of stay by at least 1 day. OR The ADR was the reason for the admission |
| Level 5 | Any level 4 ADR which requires intensive medical care |
| Level 6 | The adverse reaction caused permanent harm to the patient |
| Level 7 | The adverse reaction either directly or indirectly led to the death of the patient |
